# Probing spatial inhomogeneity of cholinergic changes in cortical state in rat

**DOI:** 10.1038/s41598-019-45826-4

**Published:** 2019-06-28

**Authors:** Tazima Nur, Shree Hari Gautam, Julie A. Stenken, Woodrow L. Shew

**Affiliations:** 1 Department of Physics, University of Arkansas, Fayetteville, AR 72701 USA; 2 Graduate Program in Microelectronics and Photonics, University of Arkansas, Fayetteville, AR 72701 USA; 3 Department of Chemistry and Biochemistry, University of Arkansas, Fayetteville, AR 72701 USA

**Keywords:** Neural circuits, Somatosensory system

## Abstract

Acetylcholine (ACh) plays an essential role in cortical information processing. Cholinergic changes in cortical state can fundamentally change how the neurons encode sensory input and motor output. Traditionally, ACh distribution in cortex and associated changes in cortical state have been assumed to be spatially diffuse. However, recent studies demonstrate a more spatially inhomogeneous structure of cholinergic projections to cortex. Moreover, many experimental manipulations of ACh have been done at a single spatial location, which inevitably results in spatially non-uniform ACh distribution. Such non-uniform application of ACh across the spatial extent of a cortical microcircuit could have important impacts on how the firing of groups of neurons is coordinated, but this remains largely unknown. Here we describe a method for applying ACh at different spatial locations within a single cortical circuit and measuring the resulting differences in population neural activity. We use two microdialysis probes implanted at opposite ends of a microelectrode array in barrel cortex of anesthetized rats. As a demonstration of the method, we applied ACh or neostigmine in different spatial locations via the microdialysis probes while we concomitantly recorded neural activity at 32 locations with the microelectrode array. First, we show that cholinergic changes in cortical state can vary dramatically depending on where the ACh was applied. Second, we show that cholinergic changes in cortical state can vary dramatically depending on where the state-change is measured. These results suggests that previous work with single-site recordings or single-site ACh application should be interpreted with some caution, since the results could change for different spatial locations.

## Introduction

The cerebral cortex is dynamic, exhibiting dramatic changes in population neural activity depending on behavioral context. During sleep, quiet restful awake state, and anesthesia, neural populations tend to exhibit coordinated waves of synchronous firing and large amplitude local field potential (LFP) fluctuations^1–3^. In contrast, asynchronous firing and low amplitude LFP fluctuations are typical during more alert, attentive, and active behavioral conditions^4–8^. The degree of population-level synchrony is traditionally, and in this paper, referred to as the ‘cortical state’^5,9,10^.

Among the most important factors governing changes in cortical state is cholinergic neuromodulation^11^. Cholinergic neurons project from diverse nuclei to diverse structured targets in cortex^12–14^. For instance, cholinergic neurons in the basal forebrain are active during both the awake state and during random eye movement (REM) sleep, but not during slow-wave sleep^15^. REM sleep and the awake state are associated with an asynchronous cortical state, while slow-wave sleep is associated with a synchronized cortical state. Blockade of cholinergic and serotonergic signaling prevents the asynchronous cortical state^16^. Conversely, stimulation of basal forebrain tends to result in decreased LFP fluctuations^17^ and reduced correlations among spiking neurons in the cortex^4^, which suggests that the cholinergic neurons in basal forebrain promote the asynchronous cortical state. Direct application of acetylcholine (ACh) agonist carbachol also abolishes the slow oscillations of the synchronized state in cortex slices^18^.

Traditionally, both cholinergic projections and changes in cortical state have been assumed to be spatially widespread and diffuse in the cortex. This view is in part due to limitations in traditional techniques. For example, single electrode measurements preclude measuring spatial inhomogeneity, which requires multiple electrodes at multiple spatial locations. Moreover, advances in recent anatomical studies reveal that cholinergic neurons project to cortex in a spatially structured, inhomogeneous manner^13,19^. Older studies also suggest that ACh distribution varies across cortical layers^20–23^ and within layers (e.g. across whisker barrels in rat somatosensory cortex^23,24^). These facts raise basic questions. Do cortical state changes depends on the spatial location of ACh release? Are cholinergic changes in cortical state also more spatially structured and inhomogeneous than previously thought?

**Figure 1:**
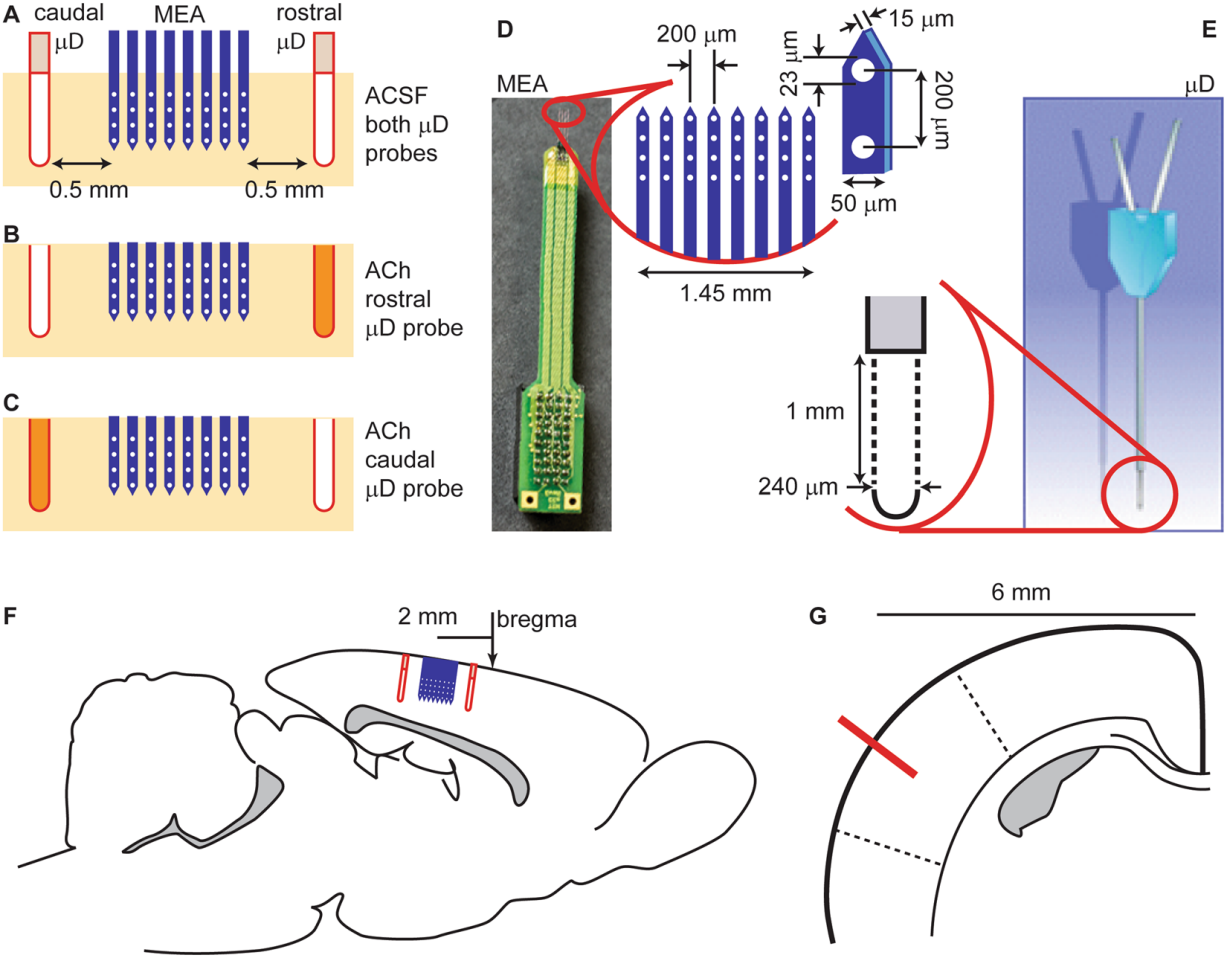
Experimental design and probe configuration. A microelectrode array (MEA) was inserted into somatosensory cortex between two microdialysis (μD) probes. Three different configurations were considered (**A**) artificial cerebral spinal fluid (ACSF) without ACh infused at both μD probes, (**B**) 100 mM ACh in rostral μD probe with ACSF in caudal probe, and (**C**) 100 mM ACh in caudal μD probe with ACSF in rostral probe. We also studied two additional cases: 1 mM neostigmine in rostral μD probe with ACSF in caudal probe, and 1 mM neostigmine in caudal μD probe with ACSF in rostral probe. (**D**) Dimensions of MEA electrodes (white circles) and shanks (blue). (**E**) Dimensions of μD membrane (dashed). (**F**) Sagital section of rat brain with the positions of the probes shown relative to bregma. (**G**) Coronal section of the rat brain 2 mm posterior to bregma. The probe location (red line) was 6 mm lateral from midline and inserted perpendicular to the cortical surface. Dashed lines indicate the approximate boundaries of barrel cortex.

To answer these questions, we require a method that can control ACh in at least two different spatial locations within the same cortical circuit. Here we describe such a method based on two microdialysis probes and a microelectrode array. We use the two microdialysis probes to create three different spatial arrangements of ACh modulation as illustrated in Fig. 1. We use the microelectrode array to measure the resulting changes in cortical state and changes in sensory response at 32 locations in barrel cortex of rats. The array is inserted such that it spans 0.6 mm depth and 1.45 mm of lateral extent within layers. The direction of insertion was normal to the brain surface. These dimensions span approximately three cortical layers (2, 3 and 4) and multiple whisker barrels, which are each approximately 0.3 mm in lateral extent. First, we show that changes in cortical state can be rather spatially inhomogeneous, both within and across layers. In extreme cases, two different spatial locations can even undergo opposite changes simultaneously - one location becoming more synchronous while the other becomes less synchronous. Second, we show that applying ACh at two different spatial locations results in dramatically different changes in cortical state.

## Results

Our first goal was to measure changes in cortical state due to changes in the spatial location of ACh application. We applied ACh at two different locations using two microdialysis (μD) probes separated by approximately 2.5 mm, each inserted perpendicular to the cortical surface to a depth of 1 mm, as illustrated in Fig. 1. Thus, the active, permeable sections of the microdialysis probes were fully submerged and nearly spanning all cortical layers. Hereafter, we will distinguish the two probes with the labels ‘rostral’ and ‘caudal’, which refer to positions rostral and caudal relative to the MEA, respectively (Fig. 1). Two different locations of ACh application were studied: (1) ACh through the rostral probe while perfusing ACSF through the caudal probe, and (2) ACSF through the rostral probe and ACh through the caudal probe (Fig. 1). We also studied a sham condition with only ACSF through both μD probes. In the results to follow, we focus primarily on recordings taken immediately following the switch from one of these patterns to another. Thus, the changes in cortical state presented are largely due to the changes in the location of ACh application.

**Figure 2:**
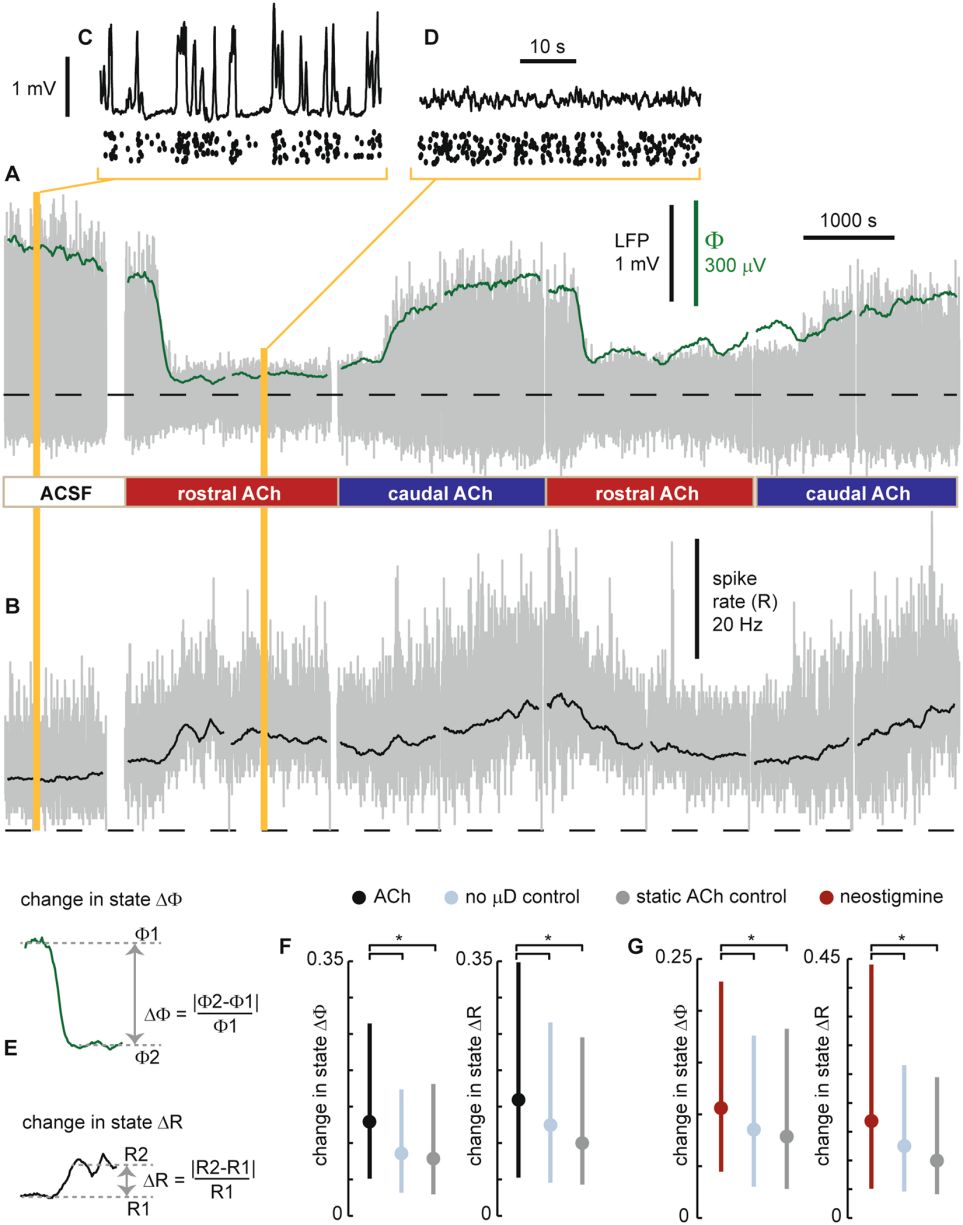
Cholinergic changes in cortical state. (**A**) Example time series of LFP from a single electrode (gray) illustrating changes in cortical state due to application of ACh at two different spatial locations. Without ACh (ACSF sham condition), the cortical state is synchronized, exhibiting large amplitude fluctuations in LFP, while the application of ACh in the rostral μD probe results in a transition to a desynchronized cortical state with small fluctuations. The entire time series is from one electrode in row 2, shank 7 (close to the rostral side of the MEA). We quantify these changes in cortical state with Φ the standard deviation of the LFP (green, computed in 120 s sliding window). Timing of switches in ACh application indicated below. Dashed line indicates zero for LFP and Φ. (**B**) Multiunit spike rate time series recorded from the same electrode used to record LFP in panel A. Gray: spike rate based on 1 s time windows. Black: spike rate based on 120 s sliding window (same as used to compute Φ). (**C**) LFP (top) and MUA spike raster (bottom) shown at higher time resolution during the condition with ACSF in both μD probes. Note the large amplitude LFP fluctuations associated with a synchronized cortical state. The vertical positions of spikes in the raster were arbitrarily shifted for better visualization; all MUA spikes are from the same single electrode. (**D**) LFP (top) and MUA spike raster (bottom) shown at higher time resolution during the condition with ACh applied in the rostral μD probe. (**E**) To quantify changes in cortical state we measures ΔΦ and ΔR, absolute fractional changes in Φ and R. (**F**) Following ACh changes, both types of change in state, ΔΦ (left) and ΔR (right), were significantly greater than those observed without any μD probes in place (light blue) and greater than those observed while ACh conditions were held fixed (gray). (**G**) Following changes in neostigmine application, both types of change in state, ΔΦ (left) and ΔR (right), were significantly greater than those observed without any μD probes in place (light blue) and greater than those observed while neostigmine conditions were held fixed (gray). For panels F and G, dots indicate median, error bars indicate upper and lower quartiles, and asterisks indicate p < 0.001 (Wilcoxon rank sum test).

Between the two microdialysis probes, we inserted a 32-channel electrode array (Fig. 1). The geometry of the electrode array was a 4 × 8 grid (eight shanks, 4 electrodes per shank) with all electrodes in the same plane. The electrode array was inserted to be coplanar with the plane formed by the two microdialysis probes. The goal of our analysis was to make time-resolved measurements of cortical state at each electrode. We quantified cortical state Φ at a given electrode at time t by computing the standard deviation of LFP fluctuations in a 2 min window centered at time t. We also assessed cortical state based on the multi-unit activity (MUA) spike rate R in the same 2 min window. Our approach of using MUA spike rates and LFP fluctuations to assess cortical state builds on a tradition established in many previous studies^5,9,25,26^. Fig. 2A–D introduces and illustrates how we quantify changes in cortical state based on changes in LFP fluctuations and MUA spike rate. To determine how cortical state changes with time, cortical state was computed at different times t = t_1_, t_2_, t_3_, … at 10 s intervals (t_n_ = t_n−1_ + 10 s). Following a change in the spatial location of ACh application, which was imposed by switching the configuration of ACh perfusion through the two microdialysis probes, we observed clear changes in cortical state. The change in cortical state typically occurred about 5 minutes after changing the ACh configuration. This delay in the change in state is partly due to the time required for the new liquid to travel from the syringe pump to the μD probe. The delay is also affected by the time required for ACh diffusion and degradation and more complex neural network mechanisms to reach a steady-state. The changes observed based on LFP fluctuations were sometimes correlated with MUA spike rate changes and sometimes anticorrelated (for example, see Fig. 2). In addition to ACh application, we studied the effects of neostigmine application using the same dual μD probe set up. Neostigmine is a reversible acetylcholinesterase inhibitor. Thus, both ACh application and neostigmine application are likely to cause increases in ACh. The goal for the experiments using neostigmine was to create increases in ACh that are closer to naturally occurring levels. Indeed, the ACh application in our experiments is likely to result in ACh concentrations well above typical levels in cortex.

Although the changes in cortical state we observed were often quite clear (as in Fig. 2), it is also important to note that changes in cortical state occur spontaneously as well. In awake animals such changes occur due to changes in arousal as discussed in the introduction above. In anesthetized animals, spontaneous state changes are well known to occur as well, in part caused by shifts in the depth of anesthesia, which can be complex and difficult to predict. To be sure our observed changes in cortical state were significant, we next compared Φ changes following ACh application to Φ changes that occur spontaneously without any ACh changes. We compared to two control conditions. In the first control condition (labeled ‘static ACh control’ in Fig. 2F,G), we measured changes in Φ during the second recording following, a change in ACh conditions (about 20–40 minutes after). This control quantifies spontaneous Φ changes keeping everything fixed except that more time has passed since the change in ACh conditions. In the second control condition (labeled ‘no μD control’ in Fig. 2F,G), no μD probes were inserted, thus providing a baseline view of how much Φ changes spontaneously with minimal experimental disturbance of the measured area. Similarly, we assessed the significance of Φ changes due to neostigmine application (Fig. 2G) and changes in R due to both ACh and neostigmine application (Fig. 2F,G right). In all these cases, we found that the changes in cortical state that occurred just after changing ACh conditions were significantly larger than those occurring in the two control conditions (p < 0.001, Wilcoxon rank sum test).

**Figure 3:**
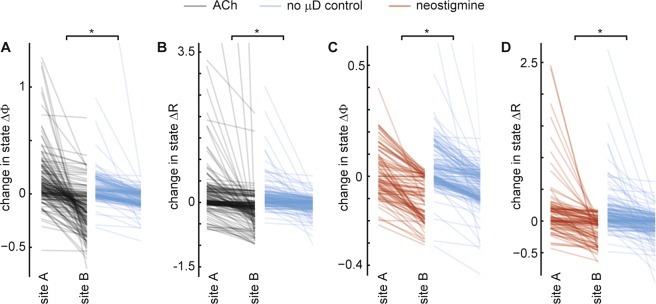
Changes in cortical state depend on spatial location of ACh manipulation. **(A**) Each gray line segment shows how the change in cortical state ΔΦ differs when ACh is applied at two different spatial locations – rostral μD probe and caudal μD probe. For clarity, site A was defined as the location which resulted in a greater ΔΦ. Each blue line segment shows how much spontaneous changes in state can be expected, obtained from a control data set with no μD probes and no ACh manipulation. Asterisk indicates that the site-dependent drops in ΔΦ are significantly greater than the control drops (p < 0.01, Wilcoxon rank sum test). (**B**) Changes in state indicated by ΔR are also significantly dependent on the ACh application site. (**C**) ΔΦ also depends significantly on the site of neostigmine application. (**C**) ΔR also depends significantly on the site of neostigmine application.

Next we sought to determine whether the changes in cortical state observed at a given electrode were dependent on which spatial location we made the change in ACh conditions (i.e. rostral or caudal μD probe). Alternatively, one might suppose that the cortical state would change in about the same way regardless of where the ACh change was made. To test these possibilities, we measured the change in state ΔΦ (and ΔR) when ACh was applied at the rostral μD probe and compared this to the change in state caused by ACh application at the caudal μD probe. We computed the absolute difference |ΔΦ_rostral_ − ΔΦ_caudal_| (and |ΔR_rostral_ − ΔR_caudal_|). These differences are shown in Fig. 3 (black and red lines), organized such that the larger of ΔΦ_rostral_ and ΔΦ_caudal_ is labeled as ‘site A’ and the smaller change is labeled ‘site B’. As a control, we computed the same absolute difference in spontaneous state change obtained at two different times during a recording with no μD probes and no ACh manipulation. Compared to this control, we found that, in all cases (ΔΦ, ΔR, ACh manipulation, and neostigmine manipulation), the changes in cortical state were significantly dependent on the spatial location of ACh manipulation (p < 0.01, Wilcoxon rank sum test).

**Figure 4:**
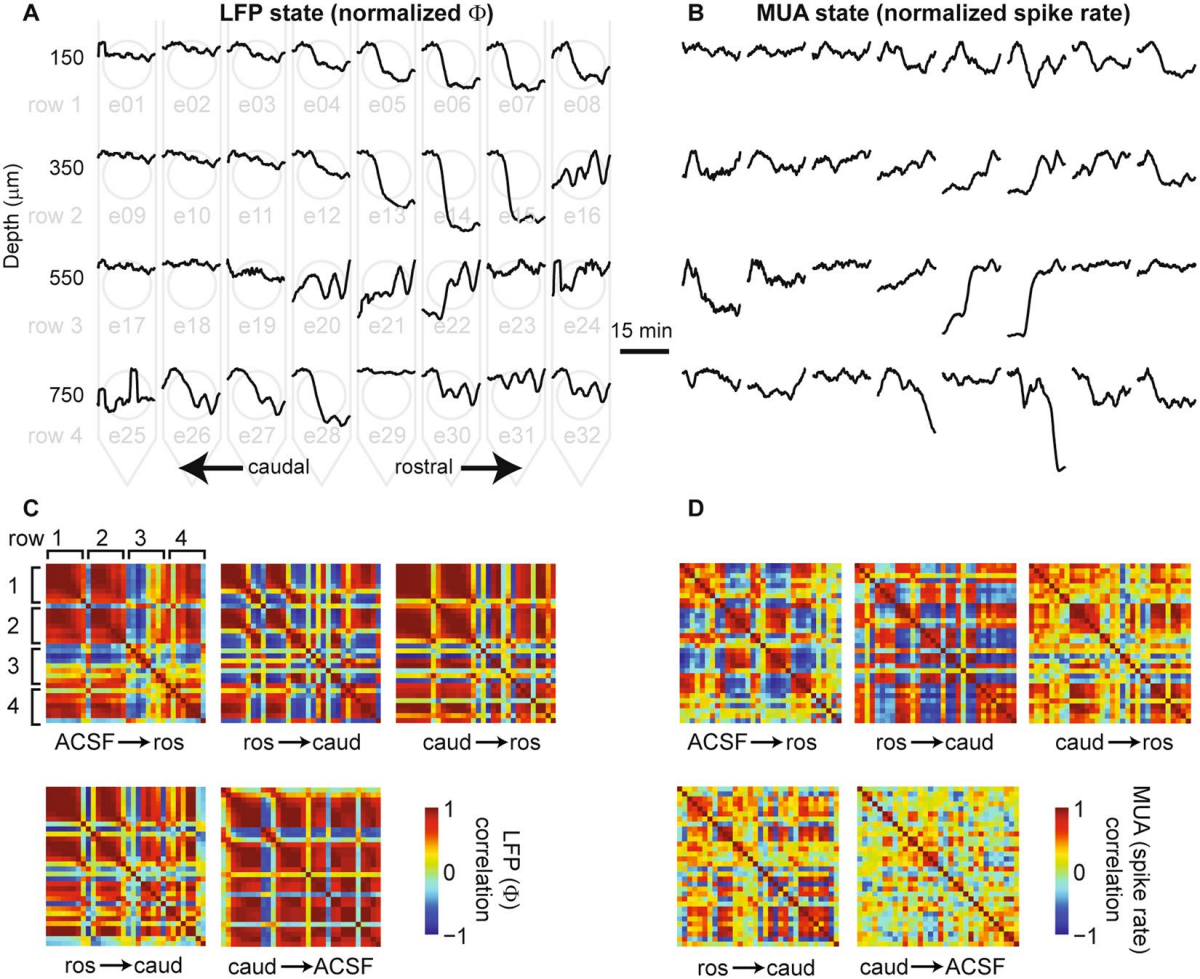
Spatial heterogeneity of cholinergic changes in cortical state. (**A**) Example of how changing cortical state Φ varies across different electrodes following the switch from the condition with ACSF only in both μD probes to the condition with ACh applied via the rostral μD probe. Each black line is a 19 min time series of Φ from one electrode, normalized by its maximum value to facilitate comparison. (19 min is the duration of one recording before switching the ACh condition again). The horizontal and vertical position of each Φ time series corresponds to the rostrocaudal position and depth, respectively, of the electrode at which the recording was made. The light gray background diagram also illustrates the arrangement of electrodes. Note that changes in cortical state Φ vary both within and across layers. (**B**) Example of how temporal changes in spike rate vary across electrodes following a switch from ACSF to rostral ACh. Data are from same recording shown in panel A. Note that the LFP-based Φ changes can differ markedly from spike rate changes. (**C**) To quantify similarity (or dissimilarity) in cortical state changes across electrodes, we computed the correlation coefficients (color code) between all possible pairs of electrodes and display them in matrix form. We show the correlation matrices for five different changes in ACh conditions (ACSF → ros indicates a switch in conditions as described in panel A, ros → caud indicates a switch from ACh application in the rostral μD probe to the caudal μD probe, etc). Note that different changes in ACh application result in quite different arrangements of which electrodes are correlated or anticorrelated. (**D**) Changes in spike rate also show diverse correlation structure for different changes in ACh application. All data in this figure came from one animal. Quantitative summaries of multiple animals are shown in Fig. 6.

**Figure 5:**
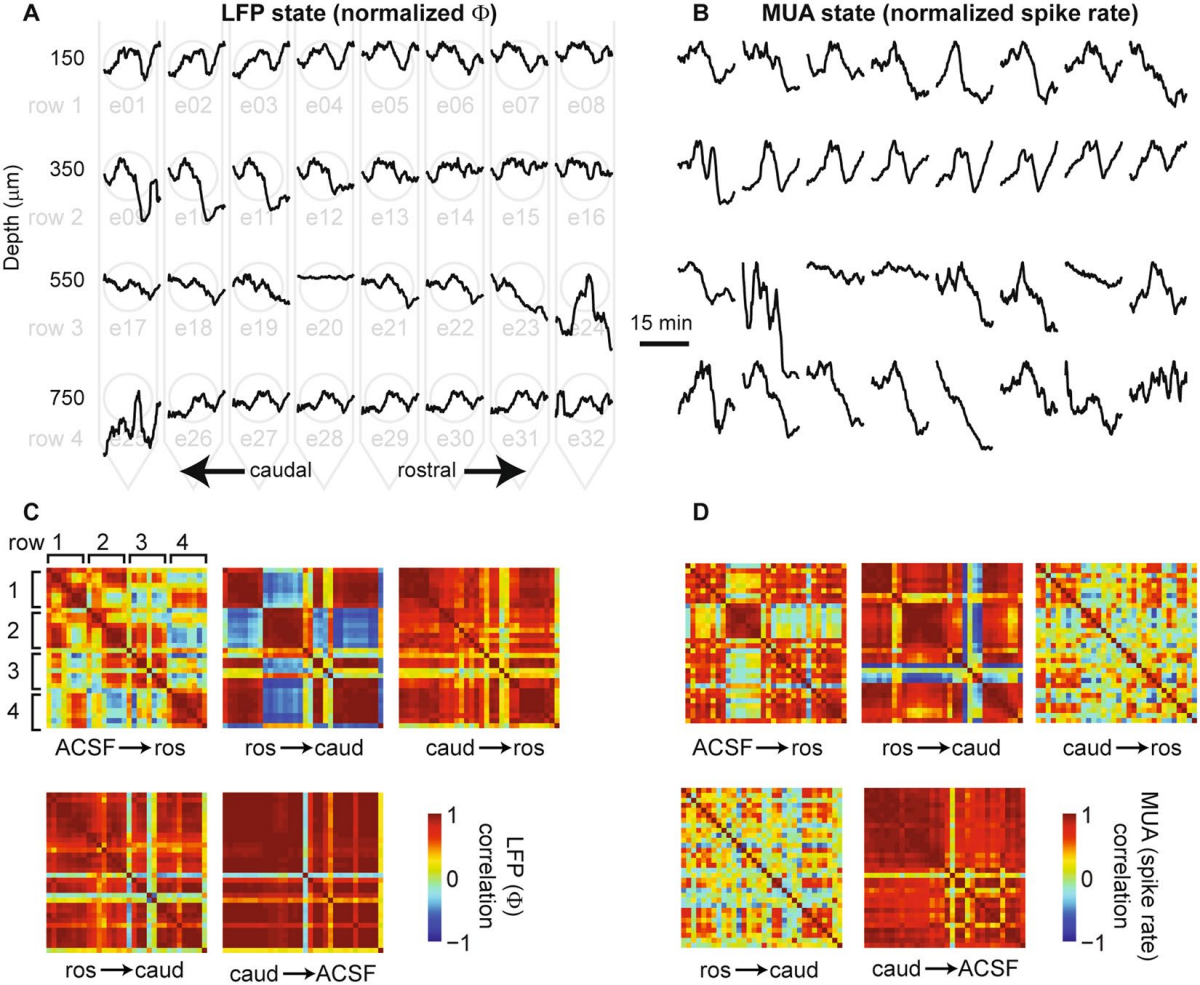
Spatial inhomogeneity of changes in cortical state due to neostigmine infusion. Interpretation of the panels of this figure are the same as for Fig. 4, except that neostigmine was applied via microdialysis probes instead of ACh. All data in this figure came from one animal. Quantitative summaries of multiple animals are shown in Fig. 6.

The evident scatter of data (Fig. 3) and the size of error bars in (Fig. 2F,G) in the above results highlight the fact that the measured change in cortical state varied substantially from trial to trial and across electrodes. Next we sought to determine the degree of heterogeneity of cortical state changes across different electrodes. Considering that it is traditionally assumed that cholinergic changes in state are spatially diffuse, we were surprised to find that changes in state could vary dramatically even at neighboring electrodes (200 μm apart) on a single trial (Fig. 4A,B). Figure 4 shows 32 time series of cortical state – one for each electrode – during the recording just after application of ACh (to the rostral μD probe in this case). Note that the data shown in Fig. 2 is the same as that shown on electrode e15 in Fig. 4A,B. To quantitatively assess the degree of heterogeneity in the state changes we observed, we computed the correlation between each possible pair of electrodes. To visualize the results, we created a pairwise correlation matrix, in which the rows and columns of the matrix are ordered according to different rows of the MEA (from shallow to deep) and color represents correlation (Fig. 4C). This revealed quantitatively that many electrode pairs were strongly correlated, while many others were strongly anticorrelated. Strong correlation indicates homogeneous state changes, while no correlation or anticorrelation indicates heterogeneous state changes. We found similar results for correlation matrices based on MUA spike rate time series (Fig. 4D). Figure 4 shows state correlations for the four types of changes studied in our experiments: (1) the initial switch from ACSF to ACh in the rostral μD probe (constant ACSF in caudal probe) labeled as ACSF → ros in the Figures), (2) switch from rostral ACh + caudal ACSF to caudal ACh + rostral ACSF (labeled as ros → caud in the Figures), (3) switch from caudal ACh + rostral ACSF to rostral ACh + caudal ACSF (labeled as caud → ros in the figures), and (4) switch from caudal ACh + rostral ACSF to ACSF in both probes (labeled as caud → ACSF in figures). Note that the switch from rostral ACh + caudal ACSF to caudal ACh + rostral ACSF was performed twice for each rat, while the other two types of changes were performed once. One correlation matrix for each of these switches is shown in Fig. 4C,D. We performed a similar analysis for the neostigmine-induced changes in cortical state (Fig. 5).

**Figure 6:**
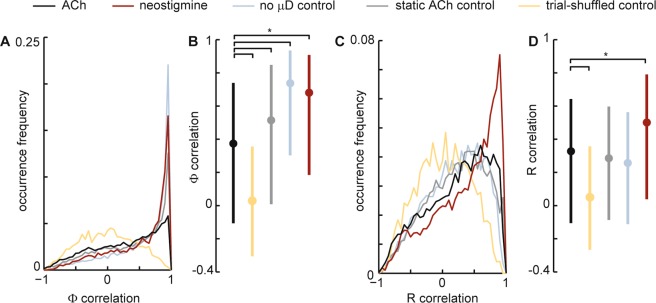
ACh application results in spatially heterogeneous changes in cortical state. (**A**) Shown are four distributions of correlation coefficients for LFP-based changes in state Φ (like those shown in Fig. 4C,D). For neostigmine application (red) and for two control data sets (no μD probes and static ACh) changes in state were rather homogeneous (many correlation coefficients near 1). For ACh application (black), the state changes were more heterogeneous, but not as heterogeneous as expected by chance (trial-shuffled control). (**B**) Same data as panel A, but shown as median (dot) and interquartile range (line). Asterisk indicates significant difference (p = 0 Wilcoxon rank sum test). (**D**) Distributions of correlation coefficients for spike rate-based changes in state R (like those shown in Fig. 5C,D. Neostigmine application resulted in the most homogeneous state changes. ACh application exhibited a similar degree of heterogeneity as two control data sets (no μD probes and static ACh), but not as heterogeneous as expected by chance.

To better gauge the observed levels of heterogeneity, we compared our results to three control data sets. The first two have been discussed above: one with no μD probes, and another with μD probes, but static ACh conditions. If our manipulations of ACh cause increased heterogeneity of cortical state, we should expect lower correlations compared to these controls. We created a third control data set to establish a baseline expectation for extreme heterogeneity. This control data set was obtained by randomizing the trial order for each electrode independently. This control should create a very low degree of homogeneity. For the LFP-based cortical state Φ, we found that ACh application resulted in a level of heterogeneity that was significantly greater than the controls with static ACh or no μD probes, but significantly less than the trial shuffled controls (Fig. 6A,B, p = 0, Wilcoxon rank sum test). In contrast, neostigmine application resulted in a higher level of homogeneity, similar to the control with no μD probes (Fig. 6A,B). For the spike rate based cortical state R, heterogeneity was generally greater than that observed for Φ. R heterogeneity was not significantly lower for ACh application compared to the controls (Fig. 6C,D). However, ACh application did cause significantly more heterogeneity than neostigmine application (Fig. 6C,D).

**Figure 7:**
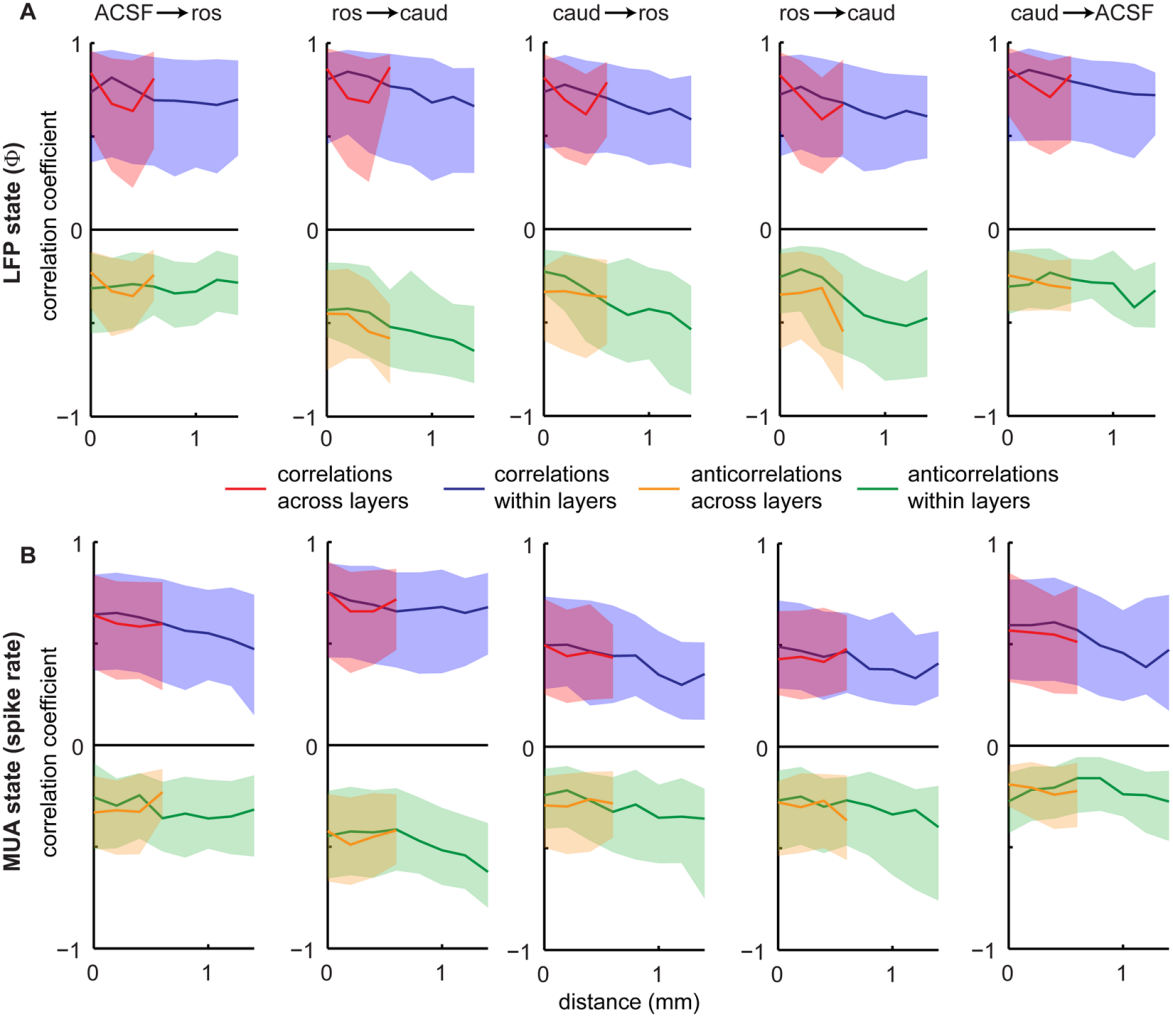
Spatial inhomogeneity of cortical state changes depends weakly on distance and direction. For all possible pairs of electrodes (n = 496) we compared the correlation coefficient of cortical state change to inter-electrode distance. Since, we observed both strong correlations and anticorrelations, we considered positive (red, blue) and negative (orange, green) correlations separately. For red and orange lines, distance across layers (i.e. inter-row distance) is represented by the horizontal axis; for blue and green lines, distance is within layer (i.e. inter-shank distance). Each subpanel contains a summary of 6 rats (n = 496 × 6 correlations). Panel A represents LFP-based changes in state; panel B represents MUA spike-rate. Shaded regions delineate quartiles, which quantify animal-to-animal variability. Solid line indicates median across animals. Distance dependent changes are small compared with variability across animals. Differences between across-layer versus within-layer changes are minimal. (ACSF → ros indicates a switch in conditions as described in panel A, ros → caud indicates a switch from ACh application in the rostral μD probe to the caudal μD probe, etc).

Finally, we sought to determine if similar state changes were more likely to occur for electrodes that are close to each other. We also wanted to determine whether similar state changes were more likely within a single row of the MEA (within cortical layer) or within a single shank (within a cortical column). To do this, we compared the correlation coefficient between two electrodes to the distance between them. We did this for two types of distance: the cross-row distance and the cross-shank distance. As shown in Fig. 7, there was a slight trend for correlations to decrease with distance, but this trend was not significantly different when comparing cross-row versus cross-shank distances. Moreover, the decrease in correlations with distance was similar in magnitude to the variability from one experiment to another. Thus, we can conclude that the spatial structure of cortical state changes are weakly distance-dependent and largely independent of direction.

## Discussion

We have described a method of combining two microdialysis probes with a microelectrode array. We used the microdialysis probes to apply ACh and neostigmine at two different spatial locations within the same cortical circuit. We found that applying ACh at different locations can cause spatially diverse changes in cortical state as measured with the electrode array. Changes in the spatial location of ACh application within a cortical circuit can result in dramatically different changes in cortical state at different locations in the cortex. By comparison, ongoing changes in cortical state with no direct manipulation of ACh are more spatially homogeneous.

One direct implication of our study is that caution is appropriate when interpreting experiments based on ACh or neostigmine that is applied at a single location. Also, it is prudent to cautiously interpret results based on single electrode recordings. Indeed, in both these cases, our findings suggest that different results could be found if the electrode is moved slightly or the ACh application site is moved slightly.

There are interesting, but less direct implications when it comes to understanding cholinergic modulation of cortical networks, in general. Cholinergic modulation plays a key role in the sleep-wake cycle as well as changes in alertness during the awake state. Moreover, in Alzheimer’s disease the loss of cholinergic neurons is thought to play an important role in dysfunction. Our findings suggest that cholinergic changes in cortical state can be much more spatially varied than traditionally expected. Whether normal, healthy cholinergic modulation of cortical circuits is as spatially varied as in our experimental paradigm remains unknown. In the case where cholinergic modulation is spatially structured, interesting questions arise. For instance, how does a spatially heterogeneous cortical state impact the ability of the cortex to encode sensory input, for example? Previous studies have found that response to sensory stimulation can vary depending on cortical state^4,5,18,27,28^. Considered together with spatially heterogeneous cortical state, state-dependent coding implies spatially heterogeneous coding. Different parts of the cortical network could be utilizing different coding strategies to interpret the same sensory input. If these different coding strategies extract different information from the same input, the spatial heterogeneity could be beneficial. On the other hand, if loss of cholinergic neurons in diseases like Alzheimer’s results in spatially inhomogeneous cholinergic modulation, this would suggest a harmful effect of spatial heterogeneity.

In addition to testing these intriguing ideas about coding and disease-related dysfunction, future studies could clarify several more basic unanswered questions raised by our results. For instance, additional experiments which measure ACh concentration directly (not just apply ACh) using our microdialysis system would clarify the extent to which cholinesterases are degrading ACh in the cortical circuits we study. Control experiments with ACh + neostigmine applied together would also help determine the influence of cholinesterases. Another interesting future experiment would be to apply cholinesterases or other anticholinergic agents to clarify the effects of cholinergic hypofunction. Such reduction of cholinergic input to cortical circuits may play an important role in Alzheimer’s disease, which is associated with the loss of cholinergic neurons.

Although our results clearly demonstrate that spatially varied changes in cortical state are possible, another limitation of our experiments is that they do not yet reveal the mechanisms of these effects.What mechanisms could explain the spatial diversity of state changes we present here? As a specific example, how is it possible that when we apply ACh at one end of our electrode array, we sometimes see the largest changes in state near the center of the array as shown in the example in Fig. 4? One would naively expect the largest changes closest to the ACh source. Similar examples were not unusual in our experiments. Although, new experiments would be required to definitively identify the mechanisms responsible for these spatially nonuniform state changes, we propose some possibilities here. One possibility is that the density of ACh receptors across mesoscale cortical networks (i.e. across a few mm) is spatially nonuniform. In this view, our specific example in Fig. 4 could be explained by a higher density of ACh receptors near the center of the array. Given the highly non-uniform spatial structure of axonal arborizations of cholinergic neurons that project to cortex^19^, it is plausible that the receptors which receive this input to cortex could be similarly spatially structured. Another possibility is that, even with spatially uniform ACh receptors, competitive mechanisms such as lateral inhibition could result in spatially non-uniform changes in state. Such spatial symmetry-breaking mechanisms are not uncommon in theoretical studies (e.g. ‘bump attractors’)^29^.

We anticipate that future studies could combine the methods we describe here with measurements of response to sensory stimulation to answer these questions.

## Materials and Methods

The experiments were designed and performed following the guidelines provided by the National Institute of Health for the Care and Use of Laboratory Animals. All the procedures were approved by the Institutional Animal Care and Use Committee (IACUC protocol # 15017) of the University of Arkansas. The primary experiments were performed on n = 8 adult male Sprague Dawley rats (average weight 357 ± 84 g, Rattus Norvegicus, Harlan Laboratories); control experiments were done on five additional rats (average weight 329 ± 93 g). Anesthesia was induced by isoflurane inhalation and maintained by intraperitoneal injection (ip) of urethane (1200 mg/kg body weight (bw) dissolved in saline). A craniotomy (3 × 6 mm) was performed over somatosensory barrel cortex (centered 2 mm posterior from bregma and 6 mm lateral from midline). Two microdialysis probes (240 µm diameter, 1 mm membrane length, CMA 11, Harvard Laboratories) and a 4 × 8 microelectrode array (8 silicon shanks with 4 iridium electrodes on each shank, 200 µm inter-electrode distance, 200 µm inter-shank distance, 1 MΩ impedance at 1 kHz, A8 × 4-2 mm-200- 200-413- A32, NeuroNexus, MI, USA) were inserted in the craniotomy window. The MEA was inserted to a depth of 800 µm. The plane of electrodes of the MEA was oriented perpendicular to the brain surface and approximately parallel to the midline of the animal. One microdialysis probe (here after referred to as the “caudal” probe) was positioned 0.5 mm caudally from the caudal end of the MEA, inserted to a depth of 1 mm. The other, “rostral” microdialysis probe was located 0.5 mm rostrally from the rostral end of the MEA, also inserted to a depth of 1 mm. Care was taken not to damage blood vessels during insertion of the electrodes and microdialysis probes. Because of the somewhat unpredictable location of blood vessels, we could not use precisely the same stereotaxic coordinates for each animal. Small gel foam pieces soaked in artificial cerebral spinal fluid (ACSF) were placed on top of the exposed brain surface to prevent the brain surface from drying. An Ag/AgCl pellet was placed in the gel foams serving as the ground for the MEA measurements. Neural activity at each electrode was recorded with a sampling rate of 30 kHz (Cerebus, Blackrock Microsystems, UT, USA).

Eleven recordings, each 19 min in duration were performed on 8 rats. For all of these, the first recording was performed with only artificial cerebral spinal fluid (ACSF) infused through both microdialysis probes at a flow rate of 2 µL/min. For five of these rats, the next two datasets were collected while 100 mM ACh was infused through the rostral microdialysis probe and only ACSF was infused through the caudal microdialysis probe. This concentration of ACh was chosen consistent with previous studies^28^. The condition was reversed for the next two datasets; 100 mM acetylcholine was infused through the caudal microdialysis probe while only ACSF was infused through the rostral microdialysis probe. These four datasets were repeated once more before recording two final datasets with ACSF infusion in both microdialysis probes. For three different rats, this same sequence of recordings was performed, except that neostigmine (1 mM) was infused instead of ACh. Neostigmine is a reversible acetylcholinesterase inhibitor, and therefore should also result in increased levels of ACh when applied.

For five control rats, we performed three MEA recordings (each 20 min in duration) without any implanted microdialysis probes and, therefore, without direct manipulation of ACh conditions. These controls established how much cortical state changes occur spontaneously without any ACh manipulation and without microdialysis probe implantation.

We assessed changes in cortical state in two ways, using (1) local field potential (LFP) and (2) multi-unit activity (MUA). All data analysis was done with Matlab (Mathworks). For LFP analysis, the raw electrical recordings were band-pass filtered (0.1–100 Hz) and down sampled to 300 Hz sample rate. We used LFP to quantify the cortical state, denoted Φ, at time t by computing the standard deviation of LFP fluctuations during a time window starting at t − 60 s and ending at t + 60 s. For MUA analysis, the raw electrical recordings were band-pass filtered (300–3000 Hz) and MUA spikes were defined as fluctuations falling below −2.5 standard deviations. We used MUA spiking activity to quantify cortical state at time t by computing spike rate during a time window starting at t − 60 s and ending at t + 60 s. We computed both LFP and MUA cortical state at 100 times at 10 s intervals for each recording and each electrode.

At multiple points in the manuscript we claim that one noisy variable is significantly greater than another. In these cases, we are considering two variables that are not normally distributed and are highly variable. We used the Matlab (Mathworks) function ranksum to calculate the p-value of a Wilcoxon rank sum test, which is nonparametric and tests the null hypothesis that one variable has a greater median than the other.

## References

[1] Steriade, M. *Neuronal Substrates of Sleep and Epilepsy*. *Control*10.1017/CBO9780511541711 (Cambridge University Press, 2003).

[2] Destexhe a, Contreras D, Steriade M (1999). Spatiotemporal analysis of local field potentials and unit discharges in cat cerebral cortex during natural wake and sleep states. J. Neurosci..

[3] Gireesh E, Plenz D (2008). Neuronal avalanches organize as nested theta- and beta/gamma-oscillations during development of cortical layer 2/3. Proc. Natl. Acad. Sci. USA.

[4] Goard M, Dan Y (2009). Basal forebrain activation enhances cortical coding of natural scenes. Nat. Neurosci..

[5] Harris KD, Thiele A (2011). Cortical state and attention. Nat. Rev. Neurosci..

[6] Kozak R, Bruno JP, Sarter M (2006). Augmented prefrontal acetylcholine release during challenged attentional performance. Cereb. Cortex.

[7] Reimer J (2014). Pupil Fluctuations Track Fast Switching of Cortical States during Quiet Wakefulness. Neuron.

[8] Vinck M, Batista-Brito R, Knoblich U, Cardin JA (2015). Arousal and Locomotion Make Distinct Contributions to Cortical Activity Patterns and Visual Encoding. Neuron.

[9] Lee SH, Dan Y (2012). Neuromodulation of Brain States. Neuron.

[10] Zagha E, McCormick DA (2014). Neural control of brain state. Curr. Opin. Neurobiol..

[11] Picciotto MR, Higley MJ, Mineur YS (2012). Acetylcholine as a neuromodulator: cholinergic signaling shapes nervous system function and behavior. Neuron.

[12] Chen N, Sugihara H, Sur M (2015). An acetylcholine-activated microcircuit drives temporal dynamics of cortical activity. Nat. Neurosci..

[13] Zaborszky L (2015). Neurons in the basal forebrain project to the cortex in a complex topographic organization that reflects corticocortical connectivity patterns: an experimental study based on retrograde tracing and 3D reconstruction. Cereb. Cortex.

[14] Kim J-H (2016). Selectivity of Neuromodulatory Projections from the Basal Forebrain and Locus Ceruleus to Primary Sensory Cortices. J. Neurosci..

[15] Lee MG, Hassani OK, Alonso A, Jones BE (2005). Cholinergic basal forebrain neurons burst with theta during waking and paradoxical sleep. J. Neurosci..

[16] Vanderwolf CH, Pappas BA (1980). Reserpine Abolishes Movement-correlated atropine-resistant neocortical low voltage fast activity. Brain Res..

[17] Metherate R, Cox C, Ashe J (1992). Cellular bases of neocortical activation: modulation of neural oscillations by the nucleus basalis and endogenous acetylcholine. J. Neurosci..

[18] Favero M, Varghese G, Castro-Alamancos MA (2012). The state of somatosensory cortex during neuromodulation. J. Neurophysiol..

[19] Wu H, Williams J, Nathans J (2014). Complete morphologies of basal forebrain cholinergic neurons in the mouse. Elife.

[20] Mechawar N, Cozzari C, Descarries L (2000). Cholinergic innervation in adult rat cerebral cortex: a quantitative immunocytochemical description. J. Comp. Neurol..

[21] Kristt DA (1979). Development of neocortical circuitry: histochemical localization of acetylcholinesterase in relation to the cell layers of rat somatosensory cortex. J. Comp. Neurol..

[22] Butcher, L. L., Oh, J. D. & Woolf, N. J. *Cholinergic Function and Dysfunction*. *Progress in Brain Research* **98**, (Elsevier, 1993).

[23] Lysakowski A, Wainer BH, Bruce G, Hersh LB (1989). An atlas of the regional and laminar distribution of choline acetyltransferase immunoreactivity in rat cerebral cortex. Neuroscience.

[24] Kristt DA (1979). Somatosensory cortex: Acetylcholinesterase staining of barrel neuropil in the rat. Neurosci. Lett..

[25] Stringer, C. *et al*. Inhibitory control of correlated intrinsic variability in cortical networks. *Elife* **5** (2016). 10.7554/eLife.19695 PMC5142814 27926356

[26] Hahn G (2017). Spontaneous cortical activity is transiently poised close to criticality. PLOS Comput. Biol..

[27] Gautam SH, Hoang TT, McClanahan K, Grady SK, Shew WL (2015). Maximizing Sensory Dynamic Range by Tuning the Cortical State to Criticality. PLOS Comput. Biol..

[28] Oldford E, Castro-Alamancos MA (2003). Input-specific effects of acetylcholine on sensory and intracortical evoked responses in the ‘barrel cortex’ *in vivo*. Neuroscience.

[29] Compte A, Brunel N, Goldman-Rakic PS, Wang XJ (2000). Synaptic mechanisms and network dynamics underlying spatial working memory in a cortical network model. Cereb. Cortex.

